# Debate: Is ACE screening doing more harm than good? Beyond the ACE score – towards more personalised approaches to risk prediction

**DOI:** 10.1111/camh.70124

**Published:** 2026-09-10

**Authors:** Alan J. Meehan, Stephanie J. Lewis, Andrea Danese

**Affiliations:** ^1^ Department of Psychology, Institute of Psychiatry, Psychology & Neuroscience King's College London London UK; ^2^ Department of Child and Adolescent Psychiatry, Institute of Psychiatry, Psychology & Neuroscience King's College London London UK; ^3^ South London and Maudsley NHS Foundation Trust London UK; ^4^ Social, Genetic & Developmental Psychiatry Centre, Institute of Psychiatry, Psychology & Neuroscience King's College London London UK

**Keywords:** Adverse childhood experiences, screening, risk assessment, primary health care, trauma‐informed care

## Abstract

Adverse childhood experiences (ACEs) are strongly associated with later mental and physical health problems, prompting growing interest in ACE screening to identify vulnerable children and adults who may benefit from interventions to prevent or mitigate these outcomes. However, evidence supporting routine ACE screening in clinical practice remains limited. Although cumulative ACE scores demonstrate robust population‐level associations with health outcomes, their individual predictive accuracy is consistently poor, with substantial false‐positive and false‐negative classifications and potential for inequitable outcomes across demographic groups. Existing screening approaches also fail to capture important contextual differences among reported ACEs, as well as factors that may promote resilience. Furthermore, positive screening results are not currently linked to clearly established, evidence‐based clinical pathways, raising wider concerns around labelling stigma and missed needs. Therefore, widespread implementation of ACE screening in its current form cannot be recommended to prevent poor health. However, future approaches that move beyond cumulative adversity counts towards more nuanced, personalised and proportionate ACE measures, which also consider broader developmental, social and protective factors, have the potential to inform more personalised risk assessment following childhood adversity.

Almost three decades of epidemiological research have demonstrated robust graded associations between adverse childhood experiences (ACEs) – characterised by various forms of abuse, neglect and household dysfunction – and a range of mental and physical health problems. These experiences are common, with as many as six in ten adults globally reporting at least one ACE (Madigan et al., 2023). The emergence of ACEs as a modifiable risk factor for health problems has led many to advocate for routine screening for ACEs in paediatric and adult healthcare settings. Proponents argue that screening could reduce the incidence of ACEs through earlier detection among vulnerable youth, mitigate negative health consequences in those already exposed through more informed referral and follow‐up care, and facilitate earlier intervention and improved outcomes by systematically identifying children and adults who may benefit from additional support (Shimkhada et al., 2022). Extensive evidence of psychological, neurobiological, and hormonal mediators between childhood adversity and later health may offer tangible intervention targets, while at a population level, adoption of ACE screening may also support greater awareness of the potential harms of early adversity and promote prevention.

Based on this rationale, ACE screening initiatives have emerged worldwide. Prominent examples include universal ACE screening and risk stratification for children and adults in the US state of California through their ACE‐Aware initiative, as well as increased administration of ACE questionnaires within frontline services across England and Wales and internationally through bodies such as the World Health Organisation (Danese et al., 2024). Typically, ACE screening involves asking patients a series of yes/no questions about current or lifetime adversity to generate a score indicating the total number of different ACEs reported. Historically, a threshold of ≥4 vs 0–3 ACEs has acted as a cut‐off for identifying those at ‘high‐risk’ for poor health outcomes, although this distinction largely reflects an ad hoc decision employed within the original ACE Study (Felitti et al., 1998). Contemporary perspectives on effective screening emphasise the need to clearly define the target condition, potential benefits and harms of screening (with strong evidence to indicate that the former outweigh the latter) and course of action for a positive screening result to optimise care continuity and ensure no patient is neglected (UK NSC, 2022). However, despite their increased implementation in routine healthcare, the extent to which current ACE screening frameworks meet these criteria remains uncertain, with concerns around their individual predictive accuracy, validity in capturing heterogeneous experiences of adversity and ability to inform actionable clinical decision‐making.

Although clear evidence has emerged in support of a dose–response relationship between cumulative ACE scores and a range of poor health outcomes at the population level, their ability to accurately discriminate individuals at the level needed to guide clinical decisions has not been proven. Indeed, the original ACEs questionnaire was not intended for individual risk prediction, and when these data have been revisited with this in mind, both the traditional ‘high‐risk’ cut‐off (≥4 vs 0–3 ACEs) and continuous ACE score yield relatively poor estimates of predictive accuracy for health outcomes (Meehan Baldwin Lewis MacLeod & Danese^2021^). Similarly, recent empirical examinations in large longitudinal birth cohorts have demonstrated poor individual prediction of mental health risk based on the ‘high‐risk’ cut‐off for both the cumulative ACE score and individual childhood adversities, despite clear differences in population‐level averages for later mental health outcomes between those reporting high vs low adversity exposure (Baldwin et al., 2021; Gondek et al., 2023).

A consistent observation across recent studies is the measure's low positive predictive value (most individuals in the ‘high‐risk’ category did not exhibit poor health outcomes) and sensitivity (most individuals with poor health outcomes were classified as ‘low‐risk’), suggesting that screening would overestimate levels of ill‐health among those with a high number of ACEs while simultaneously failing to identify the majority of those at risk. This pattern is exacerbated in studies that have examined gender and ethnic differences in mental health risk, where both ≥4 and ≥2 ACE cut‐offs generated a disproportionate number of false positives in Black male adolescents and false negatives in Hispanic females, suggesting the potential for ACEs screening to widen existing inequities in referral and follow‐up within healthcare practice (Cohen and Choi, 2022).

Related to poor predictive accuracy is concern about the validity of existing ACE measures in adequately capturing histories of adversity. Developmental pathways following adversity are heterogeneous: While many children exposed to multiple ACEs will experience health problems, many others with similar ACE scores will not. Although some clinicians use the ACE questionnaire to support an enhanced assessment to obtain more detail on the specific experiences reported (e.g. developmental timing, frequency, duration, severity), an ACE score itself does not capture this contextual information. Instead, the score assigns equal weight to a diverse range of ACEs and sums these in a simple count, inevitably sacrificing much of the complexity needed to accurately estimate a child's likely developmental trajectory. As an example, a score of 4 could be obtained for one child through a history of emotional, physical and sexual abuse and physical neglect, and for another through exposure to parental divorce and household mental illness, substance misuse and incarceration, while failing to recognise that these two children are likely to require different types of interventions (Danese et al., 2024).

Furthermore, common ACE measures do not capture all forms of adversity nor do they capture protective factors. In particular, adversities such as peer victimisation, community violence, discrimination and poverty are often not included. Failure to consider these adversities, many of which have been more salient in people from minoritised ethnic groups, may introduce further opportunities for systematic bias during screening (Cohen and Choi, 2022). Individual differences in responses and trajectories following adversity are also influenced by a variety of factors that act as sources of resilience to mitigate potential negative effects, including (among others) the characteristics of the adversity itself, genetic susceptibility, subsequent life experiences, the presence of supportive relationships and wider environmental conditions (e.g. socioeconomic and community resources). However, current ACE questionnaires do not assess these components of resilience, further diminishing their potential to capture heterogeneity in individual outcomes.

Even if a more valid ACE screener with adequate individual risk classification were developed, an effective screening programme should guide an appropriate and actionable clinical response following positive identification of ‘high‐risk’ ACE status, such as additional assessment and referral to a defined follow‐up intervention. However, the current ACE score does not yield a clinical diagnosis, nor have such evidence‐based care pathways following positive ACE screening been established (McLennan Gonzalez MacMillan & Afifi, 2024). In the absence of any systematic follow‐up, or where sufficient resources are not available in healthcare or community services, labelling patients as ‘high‐risk’ creates the potential for harm – for example, through contributing to stigma – without significant benefit (Austin et al., 2024).

Based on the summarised evidence base, it is not possible to recommend widespread implementation of ACE screening practices in their current form to prevent poor health outcomes. However, there are clear future directions towards improving detection and prevention of childhood adversity and its sequalae. First, adopting more nuanced and holistic assessment and prediction tools that move beyond simply counting ACEs to better capture wider contextual characteristics and their relationship to risk and protective factors across multiple domains is likely to enhance individual risk estimation. Second, in the absence of robust evidence‐based interventions for ACEs – and recognising that children with identical ACE scores may require very different care strategies – existing interventions for specific health outcomes could be evaluated in ACE‐exposed young people and, where necessary, modified to maximise their effectiveness within these specific populations (McLennan Gonzalez MacMillan & Afifi 2024). Third, adopting a trauma‐informed approach to all risk assessment may enable frontline healthcare workers and clinicians to better recognise and respond to ACEs and their sequelae without needing to classify individual patients. Finally, as ACEs are largely preventable, reducing their incidence may alleviate the need for additional screening. Although evidence for effective parent‐focused preventive interventions is currently limited, many of the strongest predictors of ACEs relate to social determinants of health and material resources (or lack thereof) within families. Thus, universal policies to reduce poverty and income inequality (e.g. affordable education, better employment opportunities) and caregiver burden (e.g. paid parental leave, supportive family work policies) may help mitigate population‐level exposure to childhood adversity (Madigan et al., 2023).

In conclusion, despite broad consensus that childhood adversity is a major determinant of lifelong mental and physical health, accurate ACE screening to detect risk of poor health for individual children and adults remains in its infancy. Before widespread implementation can be considered, existing ACE measures should be enhanced to improve predictive accuracy, including modifications to better capture the complexity of these experiences and heterogeneity of their health effects. Moreover, adequate assessment, referral and treatment pathways are needed to ensure that a positive screen leads to appropriate and tangible support for patients. While these goals are being pursued, clinicians should look towards more personalised and proportionate approaches to risk detection that consider adversity alongside other developmental, social and protective factors, while also embedding trauma‐informed approaches across healthcare more broadly.

## Conflict of interest statement

AD received funding from UNICEF; funding from the NIHR Biomedical Research Centre: Maudsley (NIHR203318); consulting fees from Yale University's Child Study Center and the University of Lausanne; travel costs support from ESCAP, RCPsych Faculty of Child & Adolescent Psychiatry, and Nadija Children's Hospital and Research Institute; and he is the General Secretary of the European Society for Child & Adolescent Psychiatry (ESCAP), Chair of the Advisory Committee for the Canadian Institute for Advanced Research (CIFAR) Child & Brain Development Programme, and the Academic Secretary of the Association of Child & Adolescent Mental Health (ACAMH). The other authors have declared that they have no competing or potential conflicts of interest.

## Funding information

No funding was received for the preparation of this article. SJL is supported by a Prudence Trust fellowship.

## Ethics statement

No ethical approval was required for this debate article.

## AI statement

The authors declare that they have not used AI in any capacity that would require disclosure in accordance with Wiley's AI Guidelines, nor in any capacity that would reasonably require disclosure for editors, reviewers and readers to properly evaluate their research or manuscript. The authors confirm that AI has not been used for purposes including but not limited to drafting and editing, to generate substantial text or restructure arguments, nor in the research methodology. The authors take full responsibility for the accuracy of this statement.

## Data Availability

Data sharing is not applicable to this debate article as no data were generated or analysed.
